# Alternative Hybrid Technique of Intubation Using C-MAC and Yankauer Suction Catheter: Case of A Floppy Supraglottic Mass

**DOI:** 10.4274/TJAR.2024.241651

**Published:** 2024-07-12

**Authors:** Renjith Viswanath, Sryma PB, Krishnendu S

**Affiliations:** 1 PSG Institute of Medical Sciences & Research, Department of Anaesthesiology, Tamil Nadu, India; 2 PSG Institute of Medical Sciences & Research, Department of Pulmonary, Critical Care and Sleep Medicine, Tamil Nadu, India

**Keywords:** Airway management, bougie, difficult airway, difficult intubation, endotracheal intubation

## Abstract

Supraglottic masses can be an anaesthesiologist’s nightmare due to the difficult airway scenario and bleeding risk during airway manipulation. Awake fibreoptic intubation is the primary method to secure the airway in such cases. However, most practising anaesthesiologists are not experts at handling the fibreoptic scope, especially in cases with a floppy supraglottic mass where it becomes difficult to displace the mask with the thin flexible bronchoscope. A hybrid technique of intubation in supraglottic masses using Bonfils rigid scope and C-MAC is often described but frequently not available. Here we describe a case of an elderly patient in their 80s presenting with a floppy supraglottic mass where an awake fibreoptic bronchoscope failed to secure the airway. Without access to a rigid Bonfils scope, we intuitively used a C-MAC to visualize the larynx and a yankauer suction catheter to displace the mass and perform a bougie-guided endotracheal intubation.

**Main Points**

⦁ Supraglottic masses are challenging and need individualized management for safely securing the airway.

⦁ Awake fibreoptic intubation, though gold standard in a difficult airway may not be successful in supraglottic masses due to risks of bleeding and shearing of mass into the airway.

⦁ Appropriate safety measures should be in place while dealing with intubation of supraglottic masses.

## Introduction

Awake fibreoptic intubation is the gold standard for securing the airway in difficult airway cases. Supraglottic masses can be extremely challenging to intubate with a flexible bronchoscope using awake intubation. Herein we describe a case with a floppy supraglottic mass that was handled with an alternative dual endoscopy technique using a yankauer suction catheter.

## Case Report

**Figure 1:**
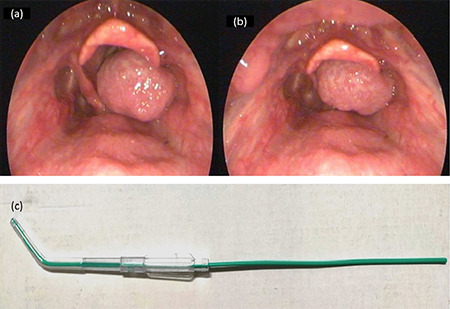
Nasoendoscopy view in inspiration showing small anterior glottic opening (a), view in expiration with mass blocking the entire glottis (b), Yankauer suction catheter with pediatric gum elastic bougie inside. The suction tip was used to displace the mass and direct the bougie into the trachea (c)

A patient in their 80s, a smoker, presented with hoarseness of voice and episodic noisy breathing for 2 months. There was no history of cough, dyspnea or hemoptysis. There was no neck swelling. Airway examination revealed Mallampati class III, normal mouth opening and neck movements. Awake nasoendoscopy showed a floppy supraglottic mass which completely covered the glottic opening with each expiration and showed a small opening during inspiration (Figure 1). There was no vocal cord palsy. Biopsy was planned under general anaesthesia with consent for emergency tracheostomy.

The patient received 1 g paracetamol intravenous, 50 µg dexmedetomidine over 30 minutes, glycopyrrolate 0.2 mg injection and nebulization with 5 mL of 4% lignocaine hydrochloride topical solution via a nebulizer and face mask over 15 minutes, starting 30 minutes before intubation. The oropharynx was topically anaesthetized with four puffs of 10% lignocaine. The ENT surgeon marked the tracheostomy site and was on standby in case of any need for a surgical airway. The patient was started on oxygen at 4LPM using nasal prongs which was continued till intubation. Awake fibreoptic intubation with a 4 mm bronchoscope showed the mass completely obstructing the glottis with expiration, part of the left vocal cord was visible with inspiration.

Displacing the mass with the flexible bronchoscope was attempted but the floppy mass kept falling into the glottis. A modified hybrid technique was tried. Propofol target controlled infusion was started and fentanyl 50 µg bolus was given. Under C-MAC vision, a Yankauer suction catheter was used to gently displace the mass to the right side, pediatric gum elastic bougie was introduced through the yankauer catheter and directed into the glottis by Seldinger technique. The setup of the Yankauer suction catheter with bougie inside is depicted in Figure 1. Yankauer catheter was removed over the bougie and reintroduced to keep the mass displaced to the right side, held in place by the assistant operator while being guided by the C-MAC view. A flexometallic tube of size 6 mm was railroaded over the bougie. Surgery proceeded with resection of the mass. The patient was extubated on the table. Histopathology of resected specimen showed moderately differentiated squamous cell carcinoma.

## Discussion

This case represents a challenging scenario where the intuitive use of the Yankauer suction catheter avoided a tracheostomy by facilitating intubation. Several safety measures were in place during the procedure. These included avoiding multiple flexible bronchoscopy attempts to reduce the chances of bleeding, carefully displacing the mass laterally before passage of the endotracheal tube to avoid shearing the mass into the airway to avoid a catastrophic complete airway obstruction, choosing a smaller size endotracheal tube and keeping the patient ready for tracheostomy before attempting to secure the airway. The use of neuromuscular blockers was avoided due to fear of mass falling into the glottis causing complete airway obstruction and inability to ventilate.

Manipulating the flexible bronchoscope is challenging in such cases. Navigating the thin flexible bronchoscope through vocal cords with a floppy supraglottic mass may need multiple attempts due to the inability to displace the mass effectively with the thin scope. This can lead to risks of bleeding, and losing the airway. Even if navigating the scope into the glottis is successful, railroading the endotracheal tube over the scope could potentially cause shearing of the mass into the airway and bleeding. The benefit of using a C-MAC in this scenario ensures complete visualization of the mass along with the tip of the endotracheal tube during intubation which is impossible while intubating with a flexible bronchoscope.

Hybrid technique refers to using two devices usually a rigid or flexible endoscope and a video-laryngoscopy (VLS).^1^ Video-assisted fibreoptic intubation (VAFI) using VLS and fibreoptic bronchoscope as well as the use of VLS and rigid endoscopes like Bonfils are the usual hybrid techniques of intubation. Hybrid techniques combine the best aspects of airway management. The VLS displaces upper airway structures making endoscope insertion easier. Endoscopes act as steerable stylet in cases with an inadequate glottic view with VLS or when intubation becomes difficult with hyper-angulated blades of VLS. The availability of two different optics helps in cases with bleeding or mucus where both views become complementary in assisting intubation. VAFI has several advantages over VLS demonstrating better glottic view and intubation success rates in cases with difficult airway,^2^ however was not useful in our case due to the floppy nature of the mass. Bonfils is a rigid fibreoptic intubating endoscope with a 40-degree curved tip, which acts like a rigid optical stylet over which endotracheal tube is loaded. It is useful in such scenarios by simultaneously providing vision and the ability to displace supraglottic mass. Being a rigid scope, it can easily navigate soft tissues, lift airway structures and can be used as a method of awake intubation.^3^ Because of the limited availability of this scope, cost and the steep learning curve involved in the use of Bonfils scope,^4^ innovative solutions used in this case with necessary precautions in place can help in avoiding a surgical airway. The yankauer suction catheter mimics the Bonfils by being a rigid angulated structure through which a pediatric bougie was inserted. In our case, since the glottic view with VLS was adequate, this technique worked as a good substitute enabling smooth intubation.

## Conclusion

Though a universal algorithm for the management of difficult airway exists,^5^ at times, such an individualized approach helps to ensure optimal outcomes rather than using conventional methods. Hybrid techniques of intubation have advantages in difficult airway scenarios like supraglottic masses where awake fibre optic intubation fails.

## Ethics

**Informed Consent:** Biopsy was planned under general anaesthesia with consent for emergency tracheostomy.
